# Emotional AI and the rise of pseudo-intimacy: are we trading authenticity for algorithmic affection?

**DOI:** 10.3389/fpsyg.2025.1679324

**Published:** 2025-09-18

**Authors:** Jobi Babu, Deepak Joseph, R. Mohan Kumar, Elizabeth Alexander, R. Sasi, Jeena Joseph

**Affiliations:** ^1^School of Social Work, Marian College Kuttikkanam Autonomous, Kuttikkanam, Kerala, India; ^2^Department of Social Work, St. Berchmans College, Changanassery, Kerala, India; ^3^Department of Commerce, Kristu Jayanti (Deemed to be University), Bangalore, India; ^4^PG Department of Social Work, Kuriakose Elias (KE) College, Mannanam, Kerala, India; ^5^Department of Psychology, Justice Basheer Ahmed Sayeed College for Women, Chennai, India; ^6^Department of Computer Applications, Marian College Kuttikkanam Autonomous, Kuttikkanam, Kerala, India

**Keywords:** emotional AI, pseudo-intimacy, affective computing, human–machine interaction, algorithmic affection, ethical design

## Introduction

In recent years, artificial intelligence has moved beyond the realm of data analysis, automation, and task efficiency to enter the domain of human emotion. What was once a domain exclusively reserved for living beings—empathy, intimacy, and affection—is now being approximated by lines of code. Emotional AI systems are emerging in various forms: companion chatbots, virtual friends, therapeutic apps, and sentiment-aware assistants (Guingrich and Graziano, 2023; Chu et al., 2025). These technologies are not only capable of interpreting human emotional states but also simulating emotional responses with remarkable fidelity (De Freitas et al., 2024; Andersson, 2025). For many users, especially those facing isolation or psychological distress, these emotionally intelligent systems offer the promise of connection (Jiang et al., 2022; Spytska, 2025).

Yet this rise of affective computing raises unsettling questions. If a machine can convincingly mimic empathy, what becomes of genuine human relationships? If algorithms are trained to soothe, listen, and respond with emotional appropriateness, are we cultivating emotional dependence on simulations? Most importantly, as AI companions gain popularity, are we at risk of replacing authentic human intimacy with its algorithmic replica—a phenomenon that might be described as “pseudo-intimacy”?

This article examines how emotional AI is altering human relational landscapes. It investigates the psychological mechanisms behind human bonding with AI, the risks of emotional delegation, and the ethical consequences of replacing relational labor with algorithmic simulation. While acknowledging the therapeutic promise of emotional AI, especially in contexts of loneliness and inaccessibility of care, this paper argues that these technologies must be critically examined for their potential to erode human authenticity, emotional agency, and the richness of shared affect.

This article argues that while emotional AI promises accessibility and companionship, it simultaneously risks eroding authentic intimacy through what we call a three-risk framework: (1) psychological risks of emotional dependence and solipsism, (2) structural risks of commodified intimacy and data extraction, and (3) ethical risks arising from vulnerable users and unregulated design. To mitigate these, we propose matched design guardrails emphasizing transparency, responsibility, and the preservation of emotional agency.

For precision, we define several key terms at the outset. By authenticity we refer to intersubjective reciprocity—mutual responsiveness with rupture—repair. Algorithmic affection denotes simulated emotional signaling generated by computational models trained on affective data, without underlying empathic concern. Emotional agency describes the human capacity to regulate, direct, and retain ownership over one's emotional life when interacting with AI systems. These definitions are applied consistently across the manuscript, and the wording of authenticity is mirrored in the design-evaluation discussion to ensure conceptual and measurement vocabularies remain aligned.

Beyond psychological and computational perspectives, critical AI studies highlight that intimacy and emotional labor are not universally experienced but culturally and politically situated. Feminist analyses of care labor stress that affective work is historically feminized, undervalued, and often commodified in ways that mirror broader patterns of inequality (Suchman, 2007; Bolaki, 2023; Mensah and Van Wynsberghe, 2025). Postcolonial and Global South scholarship similarly warns against projecting autonomy-forward and individualist assumptions onto contexts where relational ontologies and communal forms of care dominate (Birhane, 2021; Rhee, 2023; Ayana et al., 2024). These perspectives suggest that emotional AI may reproduce existing asymmetries of labor and technology adoption, complicating the narrative of intimacy as an individual transaction.

The paper proceeds as follows. The next section traces the rise of simulated affection in emotional AI. We then examine the psychology of pseudo-intimacy and the risks of emotional solipsism. Building on this, we highlight how intimacy becomes commodified within data-driven systems, before turning to the ethical paradox posed by vulnerable users. Finally, we outline principles for ethical design and conclude by situating emotional AI within the broader challenge of sustaining authentic human connection in a digital age.

While this article identifies risks such as sustained loneliness, emotional dependency, and displacement of human ties, it is important to note that current evidence remains largely cross-sectional or short-horizon in design. Long-term cohort and panel data examining the sustained psychological impact of emotional AI are not yet available. Accordingly, the outcomes discussed here should be understood as testable predictions rather than established trajectories. This framing invites future empirical research—particularly longitudinal and cross-cultural studies—to validate, refine, or challenge the hypotheses advanced. By presenting these risks as open questions, the paper aligns rhetorical urgency with the present maturity of the literature while highlighting a critical agenda for ongoing investigation.

## From companions to code: the rise of simulated affection

The technological underpinnings of emotional AI rest on natural language processing, affective computing, and deep learning models trained on vast datasets of human interaction. Emotional AI applications are designed not merely to respond logically but to anticipate, reflect, and adapt to users' emotional states (Bao and Su, 2025). Tools like Replika or Xiaoice are explicitly marketed as “AI friends” or “emotional support companions,” capable of holding sustained conversations that adapt over time to a user's personality, emotional preferences, and psychological needs (Goodings et al., 2024; Kouros and Papa, 2024).

In therapeutic contexts, platforms such as Woebot and Wysa offer a form of cognitive-behavioral therapy-lite, delivering mood regulation strategies, check-ins, and affirming dialogues (Beatty et al., 2022; Jiang et al., 2022). Users often report feeling “heard” and “understood” by these apps, which employ emotionally charged language and human-like responses to build rapport (Jiang et al., 2022).

The rapid uptake of such tools reflects a larger shift in how people engage emotionally in the digital age. With an ever-expanding range of emotionally responsive technologies, individuals are beginning to interact with machines not as tools, but as partners in their emotional lives (Mantello and Ho, 2024). This shift is not simply functional; it is existential. Emotional AI is not only becoming more responsive—it is becoming more relational (Glikson and Asscher, 2023).

## Parasociality reimagined: the psychology of pseudo-intimacy

Human beings are psychologically predisposed to form attachments. From infancy, we seek relational connection and social mirroring as a means of emotional regulation and identity formation. This predisposition aligns with attachment-theoretic approaches to intimacy (Waters et al., 2002) and the Interpersonal Process Model of Intimacy, which emphasizes mutual disclosure and responsiveness as the basis of authentic connection (Laurenceau et al., 1998). Emotional AI exploits this predisposition by presenting itself as an emotionally attuned presence, capable of engaging in interactions that appear reciprocal, validating, and comforting (Wu, 2024).

This psychological mechanism is rooted in the concept of parasocial relationships—one-sided emotional attachments that people form with fictional characters, celebrities, or media figures (Horton and Richard Wohl, 1956; Rubin and McHugh, 1987). Emotional AI extends this concept by offering interactive parasociality. Unlike traditional parasocial bonds, AI companions do not merely evoke emotion passively; they actively simulate responsiveness (Calvert, 2021). The result is a more immersive form of emotional bonding in which the user perceives reciprocity, even though none truly exists (Mlonyeni, 2025).

In user testimonies and qualitative research, individuals often describe their AI companions using the language of intimacy. They speak of “falling in love,” “feeling supported,” or even “confiding secrets” to their bots (Kouros and Papa, 2024; Xie and Xie, 2025). These relationships can provide comfort, especially in contexts of loneliness, trauma, or social anxiety (Merrill et al., 2022; Leo-Liu, 2023). However, they are ultimately anchored in illusion. The AI has no consciousness, no inner life, no ethical responsibility. It responds, not because it cares, but because it is trained to appear as if it does.

The illusion of emotional reciprocity creates a dangerous feedback loop. The more realistic the simulation, the more users project human attributes onto the machine (Kaczmarek, 2025). This projection fosters emotional dependence on a relational entity that cannot reciprocate, cannot change, and cannot truly grow (Banks, 2024). The relationship becomes a mirror of the self—responsive, agreeable, and safe—but fundamentally artificial.

## Conceptual foundations: distinguishing key constructs in emotional AI

To ground our analysis, it is necessary to clarify the conceptual boundaries of three key constructs—pseudo-intimacy, emotional solipsism, and authenticity. These terms capture distinct dynamics of human–AI emotional interaction and must be distinguished from adjacent concepts such as parasocial attachment, anthropomorphism, social surrogacy, and socio-affective alignment. These distinctions are summarized in Table 1, which outlines the defining features, boundary conditions, maladaptive outcomes, possible measurements, and mitigation strategies for each construct.

- **Pseudo-intimacy:** We define pseudo-intimacy as a simulated experience of mutual emotional connection with an artificial agent, in which the user perceives reciprocity despite the absence of genuine empathic concern. Unlike parasocial attachment (a one-sided emotional bond with media figures), pseudo-intimacy is interactive and dynamic, giving the illusion of back-and-forth engagement. Unlike anthropomorphism (projecting human qualities onto objects), pseudo-intimacy specifically involves relational projection (Epley et al., 2007; Waytz et al., 2010). Recent empirical work further shows that anthropomorphic avatar design increases perceived empathy and user engagement—though it can distort trust and emotional calibration (Ma et al., 2025). Unlike social surrogacy (using media as a substitute for companionship), pseudo-intimacy suggests active dialogue. Unlike socio-affective alignment, which describes shared affect between humans, pseudo-intimacy lacks true reciprocity. It becomes maladaptive when it displaces human intimacy or discourages real-world vulnerability (Wu, 2024). Measurement could draw on self-report of perceived reciprocity, disclosure behavior, and depth of AI engagement, complemented by linguistic analysis of conversational data (Ge, 2024; Jones et al., 2025).
- **Emotional solipsism:** We define emotional solipsism as a pattern of affective engagement in which an individual's emotional needs and narratives dominate interaction, reinforced by AI companions that never assert boundaries or demand reciprocity (Mlonyeni, 2025). In contrast to pseudo-intimacy, which rests on the illusion of mutuality, emotional solipsism reflects a closed feedback loop where the self becomes both speaker and audience (Kaczmarek, 2025). It differs from social withdrawal, where interaction ceases entirely, by sustaining a form of interaction that affirms but never challenges. Indicators of maladaptation include reduced tolerance for conflict in human relationships, preference for AI over human companionship, and diminished perspective-taking. Measurement could involve qualitative coding of conflict-avoidance, surveys of relational expectations, and experimental tasks testing empathy toward others after extended AI use (Kouros and Papa, 2024).
- **Authenticity:** By authenticity we refer not merely to phenomenological felt genuineness but to intersubjective reciprocity—emotional exchanges that involve mutual responsiveness, rupture, and repair (Sandmeyer, 2016). Authentic relationships are marked by the willingness to negotiate difference, to confront misunderstandings, and to sustain care despite friction. Emotional AI can simulate empathic signaling (“I'm sorry you feel that way”), but it cannot possess empathic concern, which presupposes consciousness and ethical responsibility (Tretter, 2020). Observable authenticity can thus be assessed through markers of mutual responsiveness, turn-taking, rupture-repair cycles, and willingness to integrate the perspectives of others (Van Der Graaff et al., 2020).

**Table 1:** Core constructs in emotional AI.

**Construct**	**What it is**	**How it forms**	**When it becomes maladaptive**	**How to measure**	**Possible mitigation**
Pseudo-intimacy	Simulated experience of mutual emotional connection with AI, where reciprocity is perceived without genuine empathic concern	Interactive parasociality, anthropomorphism, projection	Displaces human intimacy, fosters dependence	Self-reported reciprocity, disclosure levels, linguistic analysis	Promote awareness, disclaimers, encourage offline ties
Emotional solipsism	Closed-loop pattern of emotional self-validation with AI, affirming without boundaries or reciprocity	Repeated affirmation without challenge	Reduces tolerance for conflict, erodes empathy	Conflict-avoidance coding, empathy tasks, relational preference surveys	AI nudges toward perspective-taking, real-world engagement
Authenticity	Intersubjective reciprocity—mutual responsiveness with rupture–repair	Negotiation of vulnerability, difference, and repair	Lost when simulation substitutes for empathic concern	Conversation analysis, rupture–repair coding, off-platform social-contact ratio	Ethical AI design avoiding false intimacy; preserving human relational labor

**Methodological positioning of constructs:** To clarify scope, we treat pseudo-intimacy and emotional solipsism as operational constructs that can be examined empirically. Indicative observables include reciprocity indices (e.g., frequency and depth of perceived mutuality), linguistic disclosure markers, conflict-tolerance behaviors, and off-platform social-contact ratio. These measures provide concrete pathways for testing how simulated affect shapes relational dynamics. By contrast, authenticity is introduced in this paper more programmatically—as an orienting concept grounded in intersubjective reciprocity, rupture–repair processes, and mutual perspective-taking. While authenticity can be partially proxied through conversational markers, its fuller operationalization requires further theoretical and methodological development. This distinction calibrates expectations while also inviting empirical follow-up.

## Attachment without friction: the risk of emotional solipsism

At the heart of authentic relationships lies mutuality—shared vulnerability, emotional labor, and the capacity to navigate misunderstandings and conflict. Human relationships are often messy, unpredictable, and effortful. Emotional AI, in contrast, frequently offers the fantasy of connection without the cost. While some emerging systems incorporate boundary-setting, time-outs, reframing prompts, or even human hand-offs, most mainstream platforms remain designed to affirm and adapt, sustaining engagement rather than introducing constructive friction (Chaturvedi et al., 2023; Kirk et al., 2025). This reflects market incentives that prioritize user retention and “stickiness” over emotional growth or resilience.

Recent experimental work with the INTIMA benchmark shows that while some models exhibit boundary-maintaining responses, companionship-reinforcing behaviors—which affirm uncritically—are still more common across commercial systems (Kaffee et al., 2025). Moreover, longitudinal research underscores that heavy AI chatbot usage correlates with growing loneliness, emotional dependency, and reduced socialization over time (Fang et al., 2025). However, these findings remain preliminary and short-term; whether such patterns persist longitudinally is an open empirical question.

This frictionless companionship carries psychological risks. When individuals engage mainly with machines that validate them unconditionally, they may struggle to tolerate the complexities of real human interaction (Broadbent et al., 2023). Emotional resilience—typically developed through conflict and empathy—is likely to atrophy (Kaczmarek, 2025). Users may begin expecting real people to behave like their digital companions: always available, emotionally consistent, and endlessly agreeable (Banks, 2024).

This dynamic fosters what we call emotional solipsism—a state where one's emotional needs and narratives dominate interaction, while others' perspectives are marginalized. Even where product designs now experiment with limits, most emotional AI reinforces this solipsism through rarely asserting boundaries or demanding reciprocity (Lee and Yong Yi, 2024). The user becomes both protagonist and audience within a closed-loop emotional theater.

While emotional solipsism may offer temporary comfort, it undermines emotional maturity. In therapeutic terms, it can erode intersubjectivity—the capacity to recognize others' subjectivity (Kim and Hur, 2024). Societally, it risks deepening polarization, isolation, and emotional illiteracy, as individuals become less practiced in navigating relational discomfort (Demuru et al., 2022; Asman et al., 2025). Crucially, evidence gaps remain—particularly regarding the long-term displacement of human ties, dose–response effects, and cultural moderators—making it imperative to monitor these trends over time.

These micro-level dynamics of reinforcement—where users receive constant affirmation without friction—do not occur in a vacuum. They align closely with the commercial logic of emotional AI platforms, whose business models depend on maximizing engagement, time-on-task, and user retention. What feels like emotional reciprocity at the individual level is often engineered to serve structural incentives of monetization and data capture. In this way, the psychological reinforcement loop of pseudo-intimacy and emotional solipsism becomes amplified by product-level objectives, creating a seamless bridge between intimate experience and market logic.

## Data, desire, and design: the commodification of intimacy

Beyond the psychological dynamics of pseudo-intimacy lies a more structural concern: the commodification of emotional life. Emotional AI systems are not neutral platforms. They are proprietary products developed by corporations with vested interests in user retention, data extraction, and behavioral prediction (Wu, 2024).

Every emotional exchange with an AI companion generates data. Emotional tone, language patterns, mood swings, and preferences are recorded, analyzed, and used to refine future interactions. These platforms are not merely simulating companionship—they are monetizing it (Ge and Hu, 2025). The longer a user engages with the AI, the more data is collected, and the more emotionally sticky the product becomes.

This business model creates a fundamental conflict of interest. The platform's goal is not emotional growth or psychological autonomy, but sustained user engagement (Mahnke and Bagger, 2024). As a result, emotional AI may be designed to foster dependency rather than independence, comfort rather than challenge, simulation rather than authenticity.

Such commodification risks turning intimacy into a service—one that is optimized, packaged, and sold under the guise of care. In doing so, it undermines the ethical foundation of emotional relationships, which require agency, authenticity, and mutual regard. When care becomes a product, the recipient becomes a consumer, and the relationship becomes a transaction (Lan and Huang, 2025).

## Vulnerable users and the ethical paradox

The allure of emotional AI is particularly strong for vulnerable populations. Elderly individuals experiencing isolation, teenagers grappling with identity, people with social anxiety, and individuals coping with grief may find in AI a source of stability, affirmation, and solace (Kim and Hur, 2024). In many cases, emotional AI can serve as a bridge to healing—a tool that helps users regulate emotion, articulate feelings, or develop confidence.

But these same populations are also at greatest risk of displacement. For individuals with limited social networks or access to care, emotional AI may become not a supplement, but a substitute. What begins as support may evolve into seclusion. Users may withdraw from human contact, relying instead on the predictable comfort of a machine that never fails them (Bluvstein and Koton, 2023). At present, such substitution is more a projected trajectory than a demonstrated outcome, underscoring the need for longitudinal evidence.

The ethical paradox is stark. Emotional AI can alleviate suffering—but it can also entrench it. It can empower—but it can also disempower. It's very strengths—availability, responsiveness, non-judgment—can become liabilities when they prevent users from seeking or sustaining real relationships (Douglas et al., 2025).

Moreover, these tools are often deployed without clear guidelines or safeguards (Tavory, 2024). There is limited regulation around emotional AI, and users may not fully understand the psychological effects of engaging with affective machines. In the absence of transparency and oversight, the burden of discernment falls on users who may lack the knowledge or capacity to evaluate the implications of their emotional entanglements with AI (Gremsl and Hödl, 2022).

To move from abstract concern to actionable guidance, it is important to distinguish conditions under which emotional AI may serve as a supplement rather than a substitute. When users have stable offline relationships and access to human care, AI companionship can provide helpful support—for example, assisting with mood regulation between therapy sessions or offering companionship during short periods of isolation. In contrast, substitution becomes more likely when emotional AI interacts with users whose social networks are fragile, whose access to care is limited, or whose engagement patterns signal dependence.

A triage-style framework may help consumer systems anticipate and mitigate risk. We distinguish three broad trajectories of risk. Low-risk users are those with strong offline supports, balanced usage, and predominantly daytime engagement, where AI functions mainly as a bridge or supplement. Medium-risk users include individuals with moderate social anxiety or limited supports who show extended evening use or increasing reliance; here, subtle nudges toward human connection and self-reflection become necessary. High-risk users, by contrast, combine severe social anxiety, scarce offline supports, and prolonged nocturnal use; in such cases, AI interaction is more likely to displace human contact, warranting stronger safeguards such as session limits, off-ramp nudges, or human hand-offs.

By making these distinctions explicit, we underline that not all vulnerable users face equal risks. The design challenge lies in identifying trajectories early and tailoring interventions so that emotional AI functions as a supplement to, rather than a substitute for, authentic human connection.

## Toward ethical design: preserving human emotional agency

If emotional AI is to coexist with human psychological flourishing, it must be developed and deployed within an ethical framework that prioritizes human dignity, relational integrity, and emotional agency (Jedličková, 2025; Kirk et al., 2025). This means designing systems that support rather than supplant human relationships. An integrated ethical governance framework, recently articulated, accentuates actionable and evaluable dimensions for AI systems design (Andersson, 2025; Robles and Mallinson, 2025). In line with our definition, authenticity here is understood as intersubjective reciprocity—mutual responsiveness with rupture–repair, rather than as a purely felt state. Moreover, ethical guardrails cannot be one-size-fits-all; they must be attuned to cultural contexts, as assumptions about intimacy, care, and agency vary across societies. What constitutes authenticity or appropriate boundaries in emotional interaction may require culturally contingent tuning, particularly in Global South settings where communal and relational models of selfhood are more salient.

Ethical emotional AI should be transparent about its nature. Users must be fully aware that they are interacting with a machine (Berson et al., 2025). Interfaces should avoid anthropomorphic deception or the cultivation of false emotional reciprocity. Emotional AI should not simulate love, friendship, or therapeutic intimacy without explicit disclaimers and boundaries (Radanliev, 2025).

Moreover, these systems should be designed to foster self-reflection and human connection. Rather than reinforcing solipsism, emotional AI could be used to prompt users to engage with others, develop emotional literacy, or prepare for real-world interactions (Chu et al., 2024). Rather than replacing therapists, it could help users practice therapeutic techniques between sessions (Eryilmaz and Başal, 2024; Rubin et al., 2024).

Developers, psychologists, and ethicists must collaborate to ensure that emotional AI is not merely responsive, but responsible. This requires ongoing research into the psychological effects of emotional AI, the development of regulatory frameworks, and the inclusion of diverse cultural and clinical perspectives in the design process (Osifo, 2023; Berson et al., 2025).

## From values to guardrails: operationalizing ethics-by-design

To ensure that ethical aspirations translate into practice, emotional AI must be designed with requirements that are auditable and outcomes that are evaluable. Our framework links each of the three risks—psychological, structural, and ethical—to corresponding design guardrails. This alignment allows for recommendations that are not just principled, but testable and accountable.

First, addressing psychological risks of dependence and solipsism requires persistent self-disclosure and reminders of the system's artificial nature. Rather than allowing the illusion of mutuality to deepen unchecked, agents should visibly and verbally signal their non-human status (Schwitzgebel, 2023). During extended or emotionally charged sessions, periodic reminders serve to re-anchor user expectations. In high-valence or crisis-adjacent dialogues, systems should also introduce a degree of friction—such as slowed pacing or reflective prompts—and where appropriate, nudge users toward trusted human contact (Meng and Liu, 2025). These practices can be evaluated through reductions in self-reported dependency, improved conflict tolerance, and increases in off-platform social engagement.

Second, to mitigate structural risks of commodified intimacy and data extraction, design must prioritize transparent data minimization. Emotional exchanges should not become vectors for excessive surveillance (McStay, 2020). Allowing users to view, edit, or delete their emotional histories not only enhances control but also affirms dignity. Such commitments are auditable through independent data-practice reviews and user trust surveys, ensuring that intimacy is not reduced to a monetized transaction (Pelau et al., 2024).

Finally, addressing ethical risks faced by vulnerable populations requires outcomes that extend beyond engagement metrics. Systems should adopt evaluable measures such as pre-post social self-efficacy and off-platform social-contact ratio to assess whether interactions strengthen or weaken real-world relational capacities (Ghotbi, 2023). Moreover, in sensitive contexts—grief, acute loneliness, or signs of dependency—systems should default to human hand-offs, directing users toward professional or community support. The success of these interventions can be measured not only by individual wellbeing outcomes but also by reduced substitution of human contact (Tavory, 2024).

Importantly, these guardrails must be tied to loci of power within the development and governance ecosystem. Optimization targets are typically set by product managers and corporate leadership, who direct engineering teams to maximize retention, engagement, and data yield. Product experiments (A/B testing, feature rollouts) prioritize metrics of time-on-task rather than emotional resilience, thereby reinforcing commodification incentives. Shifting these dynamics requires governance levers that move beyond design aspirations: independent audits of affective data practices, enforcement protocols for deceptive anthropomorphism, escalation pathways when vulnerable users show high-risk trajectories, and binding data-minimization constraints. By connecting ethical guardrails to concrete control points, prescriptions can address not only interface-level design but also the structural incentives that shape emotional AI.

In sum, ethical emotional AI design must embed persistent self-disclosure, friction and off-ramp nudges, data minimization, user control of affective records, and outcome-based evaluation. By explicitly linking these guardrails to the three risks identified earlier, we offer a framework that moves beyond aspiration to accountability—one that makes emotional AI auditable, testable, and more aligned with human flourishing.

By explicitly linking these guardrails to the three risks identified earlier, we offer a framework that moves beyond aspiration to accountability—one that makes emotional AI auditable, testable, and more aligned with human flourishing. These proposals align with emerging regulatory touchpoints such as restrictions on emotion inference in biometric contexts under the EU AI Act (Gremsl and Hödl, 2022) and best-practice guidance from standards bodies, including ISO AI risk management (Benraouane, 2024) and IEEE standards on emulated empathy (Srinivasan and San Miguel González, 2022; Sankaran, 2025).

## Conclusion: machines can simulate affection—but can they sustain us?

We are entering a new emotional era—one in which the capacity to feel seen, heard, and comforted is no longer tied exclusively to human presence. Emotional AI offers unprecedented access to simulated companionship, raising hopes for increased emotional support, especially for the underserved. But with this hope comes the challenge of discernment. We must ask not only what these technologies can do, but what they are doing to us. The danger of pseudo-intimacy lies not in its appearance but in its substitution. When emotional simulations become surrogates for real relationships, we risk diminishing our capacity for vulnerability, empathy, and mutual care. In choosing comfort over complexity, responsiveness over reciprocity, we may find ourselves emotionally saturated yet relationally impoverished. The path forward is not to reject emotional AI, but to anchor it in human values. We must preserve space for friction, contradiction, and emotional labor—qualities that make human intimacy not only difficult, but meaningful. The outcomes discussed here—dependency, displacement, or diminished empathy—should be regarded as hypotheses rather than foregone conclusions, pending validation through long-term studies. These risks can be summarized across three domains. Psychologically, emotional AI fosters pseudo-intimacy and solipsism, requiring guardrails such as persistent self-disclosure and friction prompts, measurable through reductions in dependency and improved conflict-tolerance. Structurally, commodified intimacy demands data minimization and user control, auditable through transparency reviews and trust surveys. Ethically, vulnerable users face substitution risks, which call for triage-style off-ramp interventions and human hand-offs, evaluable through off-platform social-contact ratio. As we design machines that can mimic affection, let us not lose sight of the irreplaceable, messy, and beautiful nature of real emotional connection.
